# Soil nutrition and foliar intervention in *Brassica napus* (L.) to impair biological fitness of green peach aphid (*Myzus persicae* Sulzer)

**DOI:** 10.3389/finsc.2026.1853500

**Published:** 2026-06-26

**Authors:** Muhammad Wajid Javed, Waseem Akram, Muhammad Umair Gulzar, Ammara Riaz, Muhammad Sagheer, Asad Aslam, Ayman El Sabagh, Mohammed Antar

**Affiliations:** 1 Department of Entomology, University of Agriculture, Faisalabad, Pakistan; 2 Ministry of Science and Technology, Islamabad, Pakistan; 3 Department of Entomology and Food Science, University of Layyah, Layyah, Pakistan; 4 Department of Life Sciences, Khwaja Fareed University of Engineering and Information Technology, Rahimyar Khan, Pakistan; 5 Entomological Research Substation, Multan, Pakistan; 6 Faculty of Agriculture, Siirt Universitesi, Siirt, Türkiye; 7 Department of Agronomy, Faculty of Agriculture, Kafrelsheikh University, Kafr El-Shaikh, Egypt; 8 Agronomist and Independent Researcher, Windsor, ON, Canada

**Keywords:** antibiosis, compost, pest resistance, salicylic acid, silicon

## Abstract

**Introduction:**

Green peach aphid (*Myzus persicae*) can reduce yield of canola (*Brassica napus*) from 35-60% either directly by sap sucking or indirectly by virus transmission. Use of synthetic insecticides is not an ecologically acceptable option.

**Methods:**

Therefore, effectiveness of different foliar interventions based on salicylic acid-SA and citric acid-CA at 0, 0.5, and 1 mM, and soil nutrition through herbivore-deterrence (silicon-Si and ammonium sulphate-Ams at 25 and 50 kg ha^-1^) and soil amendments (elemental sulphur-ES, bio sulphur-BS, Cp-Compost, ES+Cp, and BS+Cp) were assessed against different biological fitness parameters of aphid such as aphid development period (days), reproduction time (days), progeny production (No.), immature becoming adults (No), and nymphal survival (%).

**Results:**

In foliar intervention treatments, 1 mM SA was the most effective treatment followed by 0.5 mM SA and 1 mM CA in reducing aphid biological parameters (10.21 to 71.5%), while in herbivore-deterrence and soil amendments, 50 kg Si (23.5-66.04%) and ES+BS (21.5-42.9%) performed better, respectively.

**Conclusion:**

These results are important in sustainable management of aphids, improving canola yield, and ensuring global food security. However, deciphering the underlying mechanism of pest resistance in canola should be an important goal of future research.

## 1 Introduction

The third most popular oilseed crop worldwide is rapeseed or canola (*Brassica napus* L.) (1). *Myzus persicae* (Sulzer) (Hemiptera: Aphididae), also known as green peach aphid or potato peach aphid (2), is a highly cosmopolitan and polyphagous insect pest in North America, Europe, and Asia (3). With a life cycle of 15 days, *M. persicae* winged adults reproduce parthenogenetically through a single sexual generation (4). It attacks a variety of agricultural crops both directly by sucking the cell sap to deplete nutrients in plants and indirectly through the spread of plant viruses, especially Turnip yellows virus (TuYV) which can cause 40% yield loss (3).

This pest is controlled through multiple applications of synthetic insecticides (e.g., pyrethroid, carbamate, organophosphate, and neonicotinoids) which have made it an insecticide resistant pest. Moreover, use of insecticides have also reduced the populations of honeybees and other beneficial insects (5). Insecticides may also have non-target effects on natural biocontrol agents (6, 7). Therefore, prospective environmentally friendly pest control methods that allow for higher crop yields with minimal threat to non-target organisms are receiving more attention (2, 8).

Use of soil amendments, including sulphur and compost, are relatively new pest management techniques, which are environmentally safe, socially acceptable, and economically cost effective (9). By administering different dosages of salicylic acid (SA), jasmonic acid (JA), and citric acid (CA), agricultural crops activate their natural defenses against biotic and abiotic stressors (10). Besides reducing abiotic stress (11), CA has demonstrated the ability to manage plant stress along with SA which can also activate insect resistance against aphids and other insects in a variety of ways such as making the plants as less preferable host, triggering harmful chemicals in plants to affect food digestion, and reducing insect biological fitness (12, 13).

Similar mechanisms are activated in hostplants in response to crop nutrition, e.g., ammonium sulphate (Ams) and silicon (Si) are used in different crops to generate nutrient-based herbivory deterrence in plants to boost plant production and pest resistance (9). Ams has been reported to improve “systemic acquired acclimation (SAA)” in plants which is stress tolerance response in the entire plant body when any of the plant part is exposed to environmental stress (14). Also, the antibiotic effect of silicon against different canola aphids has been well reported by Abbas et al. (9). Similarly, enhancing the quantitative availability of sulphur through the application of elemental (ES) or bio sulphur (BS) to canola crop triggers biosynthesis of plant defense chemicals (glucosinolates and phenolics) in canola against insect pests (15). Compost (Cp) has been shown to enhance crop production and pest resistance (16). All these approaches cause the plants to develop “systemic acquired resistance (SAR)” when “pathogenesis-related proteins” build up in plants, to trigger plant defense against the invading herbivore especially plant pathogens (17).

The objective of the study was to assess the impact of different plant defense strengthening foliar and soil treatments on bio-fitness parameters of *M. persicae*. According to the study hypothesis, different foliar and soil treatments on *B. napus* may have an impact on reducing the biological parameters of aphid (*M. persicae*) to support their possible application in pest management. This was the first study of its nature where *M. persicae* fitness parameters were assessed on canola.

## 2 Materials and methods

### 2.1 Cultivation of plants and insect culture

*Brassica napus* plants were cultivated in screen house (temperature: 22 ± 5 °C, relative humidity: 60 ± 5%, and 10 hour light/14 hour dark conditions). A cultivar named “Faisal Canola” was sown in sterilized pots (7 kg soil mass; 23 cm diameter and 20 cm depth) having soil nitrogen 0.05%, available phosphorous 7.41 ppm, and potassium 147 ppm. Urea, Di Ammonium Phosphate (DAP), and Sulphate of Potash (SOP) were applied to provide nitrogen, phosphorous, and potassium in the soil at the rate of 90, 60, and 50 kg ha^-1^, respectively, as per recommendations (18). Plants having an age of 5 weeks were bioassayed and their positions were regularly randomized to avoid any positional effect.

*M. persicae* was collected from different host plants (*B. napus, B. oleracea*, and *B. jucea*) and reared on *B. napus* hosts for more than 20 generations. A homogeneous aphid population was prepared by placing single females from a laboratory colony in 3-cm-diam. 1.5-cm-depth clip cages. Each female was allowed to produce young ones for 24 hours and then removed. The young ones/nymphs were then maintained on individual plants till the adult stage.

### 2.2 Preparation and application of treatments

The foliar treatments were comprised of salicylic acid (SA) and citric acid (CA) at a concentration of 0, 0.5, and 1 mM, which were compared to a control (non-treated plants). These concentrations were selected on the basis of previous experiments (19). The 1 and 0.5 mM was prepared by dissolving 138.12 and 69.06 mg of SA in 1-liter solvent containing 0.1% ethanol, respectively. On similar pattern, 192.12 and 96.06 mg of CA were dissolved in 1 liter of distilled water to produce 1- and 0.5-mM concentration of CA (20). The 0 mM treatments were also followed which were actually those where concentration of either SA or CA, was zero, hence, 0.1% ethanol and water were applied for 0 mM SA and 0 mM CA, respectively. These were actually the solvents which were used to prepare different concentrations of the respective inducers. The 0 mM CA had water while 0 mM SA had 0.1% ethanol. Foliar treatments were applied manually using a top-gun sprayer at a volume of 30 ml per plant.

For herbivore-deterrence experiments, ammonium sulphate (Ams) and silicon (Si) were incorporated into the soil at a rate of 25 and 50 kg ha^-1^ using 12.5 and 25 mg of each nutrient per kg of soil mass based on previous findings (21). On the other hand, soil amendments experiments had elemental sulphur (ES), bio sulphur (BS), compost (Cp), and their mixture (ES+Cp and BS+Cp). Both sulphur formulations were mixed into the soil at a rate of 4 g per kg while compost was added at a rate of 8 g per kg of soil (22). Specifications of all treatments have been given in **Supplementary Table 1**. The control treatments were maintained under the same screen house conditions.

### 2.3 Biological fitness of *Myzus persicae*

About 10 days old, individual adult females of *M. persicae* were placed on the *adaxial* side of fully expanded middle leaves of *B. napus*. These females were confined under clip cages for 24 hours to lay offspring and then mother aphids were removed. Later on, 10 neonates (replication) per treatment were proceeded in each experiment. Each clip cage had one neonate. The time taken by each neonate from its birth until producing first nymph was referred “development period.” After that, number of neonates per aphid were counted and removed until the aphids stopped reproducing. This was marked as “reproduction time.” While the number of neonates produced by the aphid was, “progeny production” and number of aphids that reached their adult stage were called “immature becoming adults.” Nymphal survival was noted by dividing the number of alive nymphs to the total number of nymphs and then multiplying the outcome with 100. If an aphid escaped or squashed accidently then it was replaced with a new one and data was censured accordingly.

### 2.4 Statistical analyses

Data were checked for normality and homoscedasticity using Shapiro-Wilk and Bartlett test, respectively and later on, analyzed statistically using GraphPad Prism 10, Version 10.6.1 (GraphPad Software Inc., San Diego, CA, USA), if assumptions of ANOVA were met. Microsoft Excel 19 (Microsoft Inc., USA) was used to generate graphs. Data following the assumptions of normality were analyzed using One-Way ANOVA following Tukey HSD test (*p* < 0.05). Otherwise, data were transformed using the function Y=log (Y + 1) and then analyzed.

## 3 Results

### 3.1 Foliar intervention treatments

**Figure 1:**
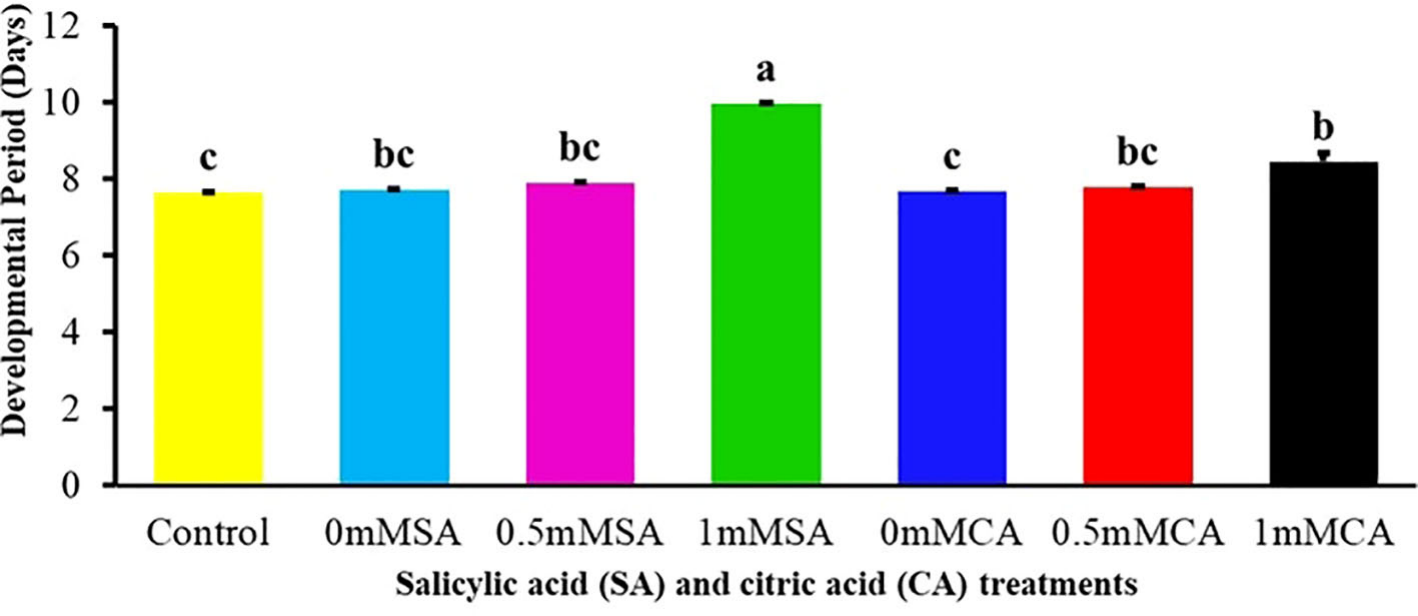
Developmental time (days) of *Myzus persicae* (Mean ± SD, *n* = 10) in foliar intervention treatments due to foliar application of salicylic acid (SA) and citric acid (CA) treatments. Different lower case letters indicate statistical significance at *p* < 0.05.

**Figure 2:**
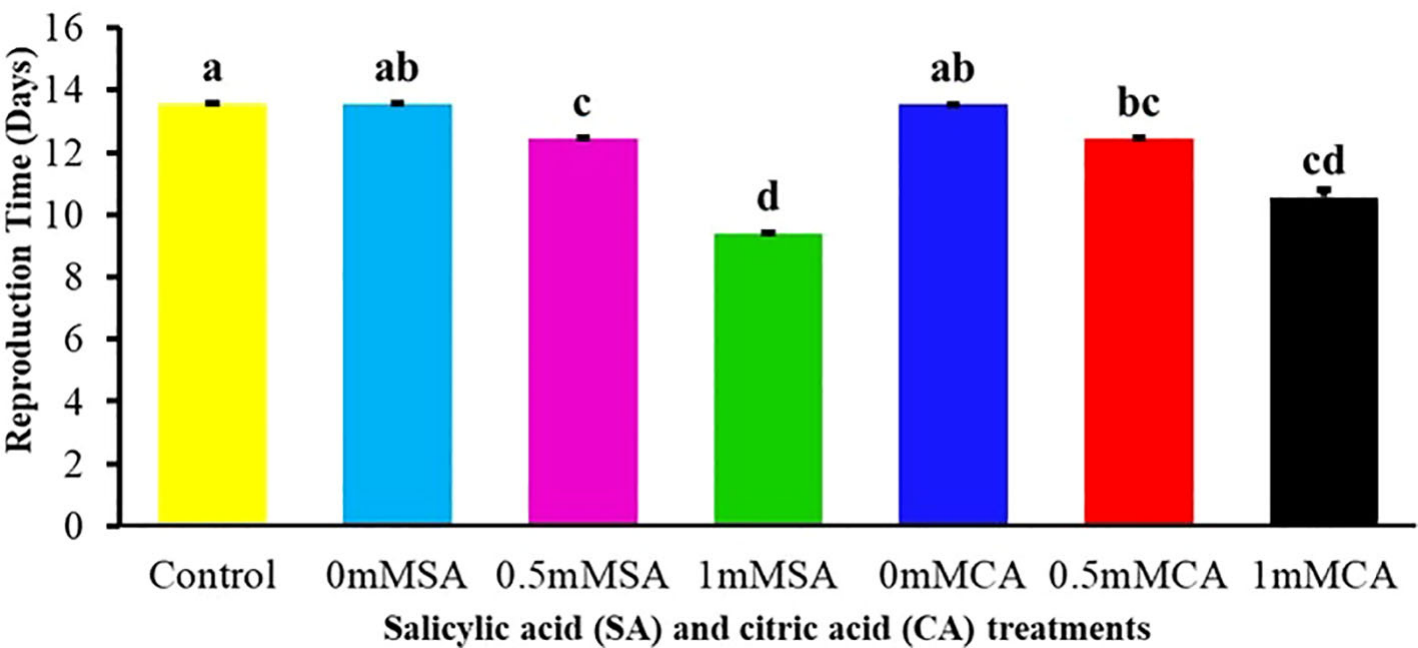
Effect of foliar applications of salicylic acid (SA) and citric acid (CA) on the reproduction time (days) of *Myzus persicae*. Data represent mean ± SD (*n* = 10). Different lowercase letters indicate statistically significant differences (*p* < 0.05) among treatments.

Results revealed that the developmental period of *M. Persicae* delayed significantly (F_6, 63_ = 15.1, *p* < 0.0001) compared to the control for both SA and CA treatments (**Figure 1**). However, in comparison to CA treatments, SA was the most effective in extending the aphid development. Development was slowed by 10.21% with 1 mM SA concentration, followed by 8.2% with 1 mM CA, and 7.51% with 0.5 mM SA. The *M. persicae* reproduction time was significantly (F_6, 63_ = 670.5, *p <* 0.0001) reduced as compared to no treatment plants (**Figure 2**). It was observed that minimum reproduction time was found in the plants treated with 1 mM SA followed by 1 mM CA which was reduced by 29.5% and 21.3%, respectively when compared to the control. Middle concentration (0.5 mM) reduced the aphid reproduction time by 8.2% (SA) and 7.98% (CA) over the non-treated controls.

**Figure 3:**
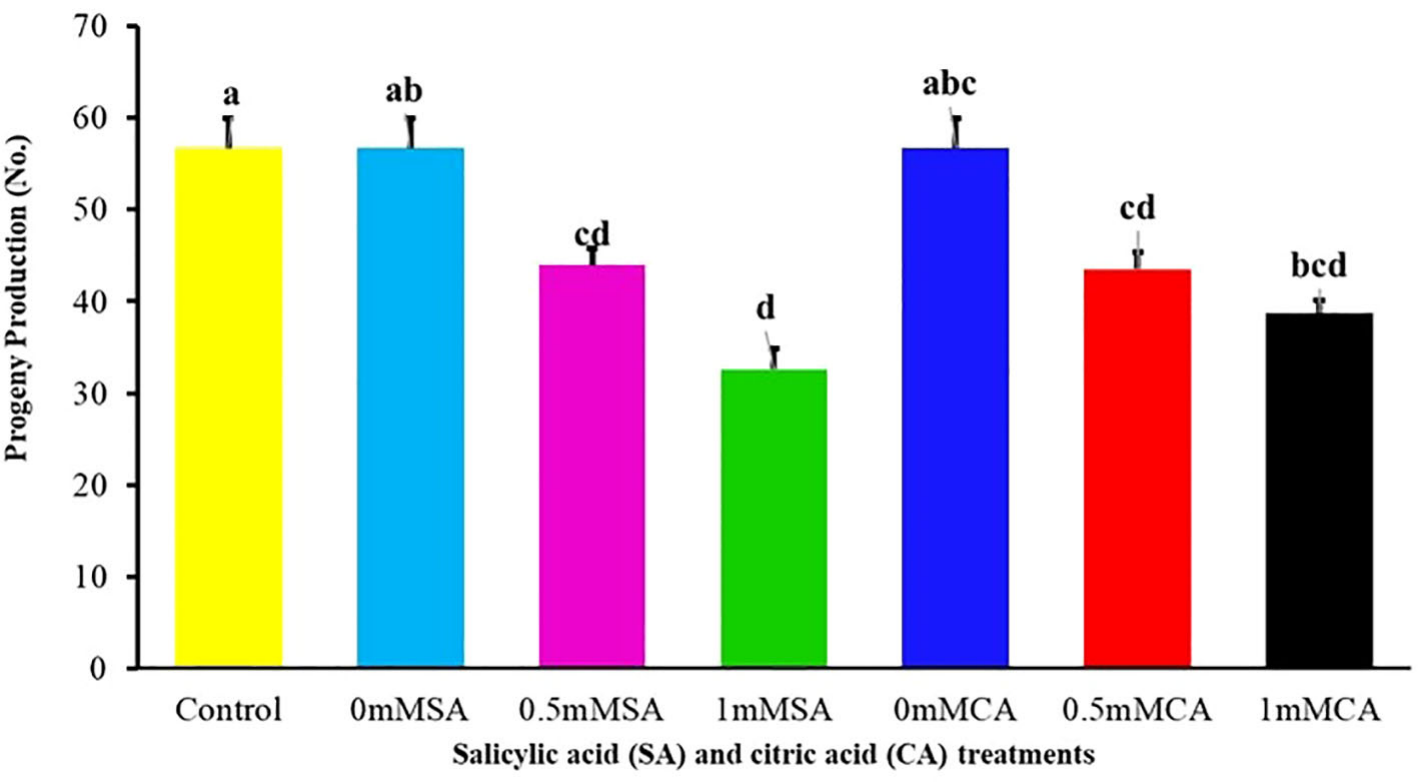
The progeny production (No.) of *Myzus persicae* following foliar application of salicylic acid (SA) and citric acid (CA) in foliar intervention treatments. Values are presented as mean ± SD (*n* = 10). Bars annotated with different lowercase letters are significantly different (*p* < 0.05).

**Figure 4:**
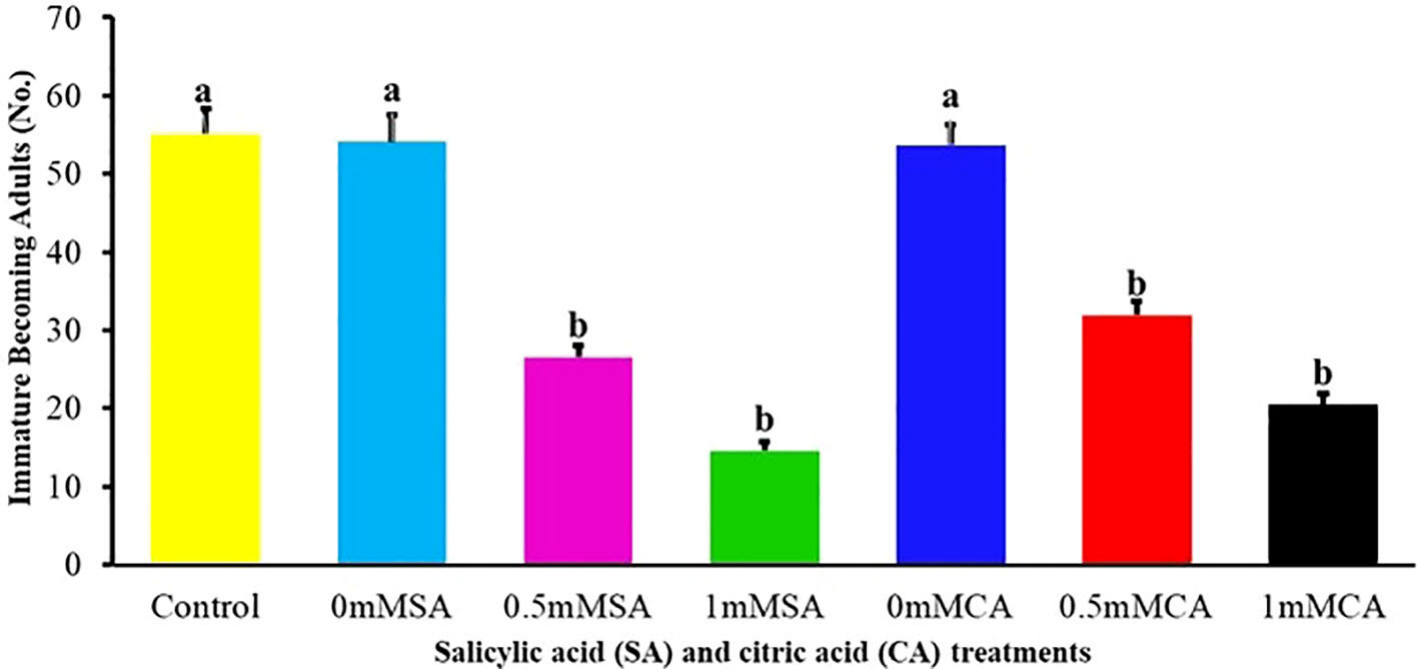
*Myzus persicae* conversion of immature into adults (No.) after foliar treatment with salicylic acid (SA) and citric acid (CA). Means (± SD, *n* = 10) with different letters differ significantly (*p* < 0.05).

**Figure 5:**
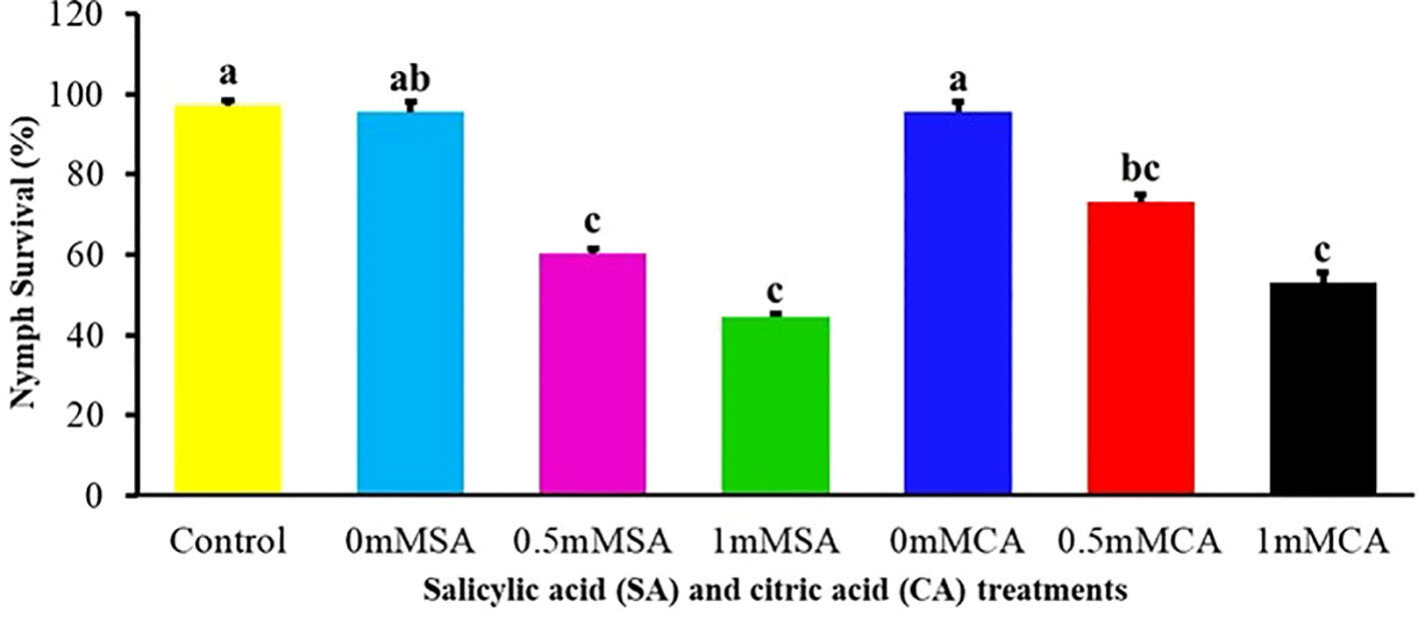
Nymph survival (%) of *Myzus persicae* under foliar intervention approach due to salicylic (SA) and citric acid (CA) treatments. Bars indicated mean values ± SD (*n* = 10) and annotated different lowercase letters showed statistical significance (*p* < 0.05).

Similarly, progeny development of aphid population was also reduced significantly (F_6, 63_ = 5.7, *p* < 0.0001) (**Figure 3**). The 1 mM SA treatment resulted in the maximum decline in progeny production (39.7%), followed by 34.1% in 1 mM CA, 23% in 0.5 mM of both SA and CA when comparison was made in case of control (no treatment). The conversion of immature aphids to adult also was found significantly (F_6, 63_ = 34.7, *p* < 0.0001) influenced by both treatments (SA and CA) (**Figure 4**). The 1 mM SA was more efficient in reducing the conversion (71.05%) as compared to control treatment, followed by 1 mM CA (64.5%). Maximum percentages of conversion to adults were observed in either control or 0 mM treated plants. The nymphal survival percentage also displayed statistically significant difference (F_6, 63_ = 54.3, *p* < 0.0001) from the control treatment (**Figure 5**). Results depicted that minimum nymph survival percentage was observed in 1 mM SA (41.1%) followed by 1 mM CA (57.5%), 0.5 mM SA (59.3%), and 0.5 mM CA (64%), while, 97.4% nymphs were survived in case of control treatment.

### 3.2 Herbivore-deterrence experiment

**Figure 6:**
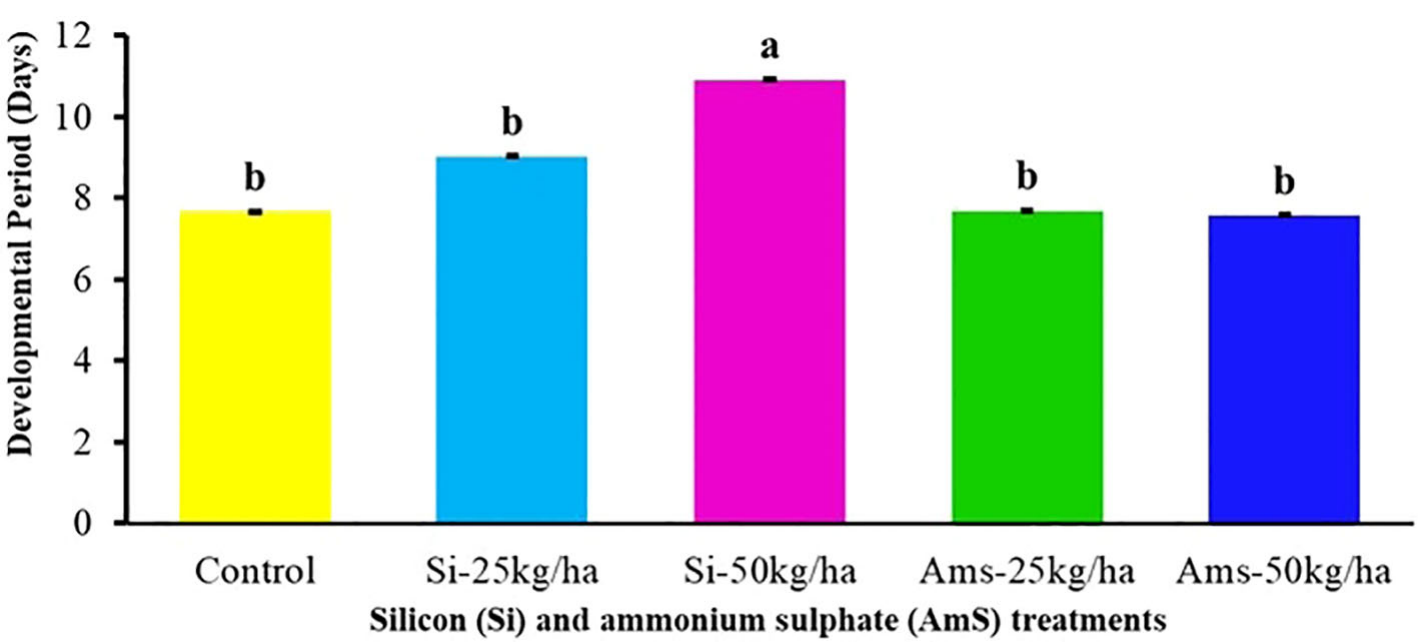
Developmental period (days) of *Myzus persicae* under herbivore-deterrence experiments due to soil-incorporated treatments of silicon (Si) and ammonium sulphate (Ams). Bars indicated the data (Means ± SD, *n* = 10) having annotated different lowercase letters (Statistical significance *p* < 0.05).

**Figure 7:**
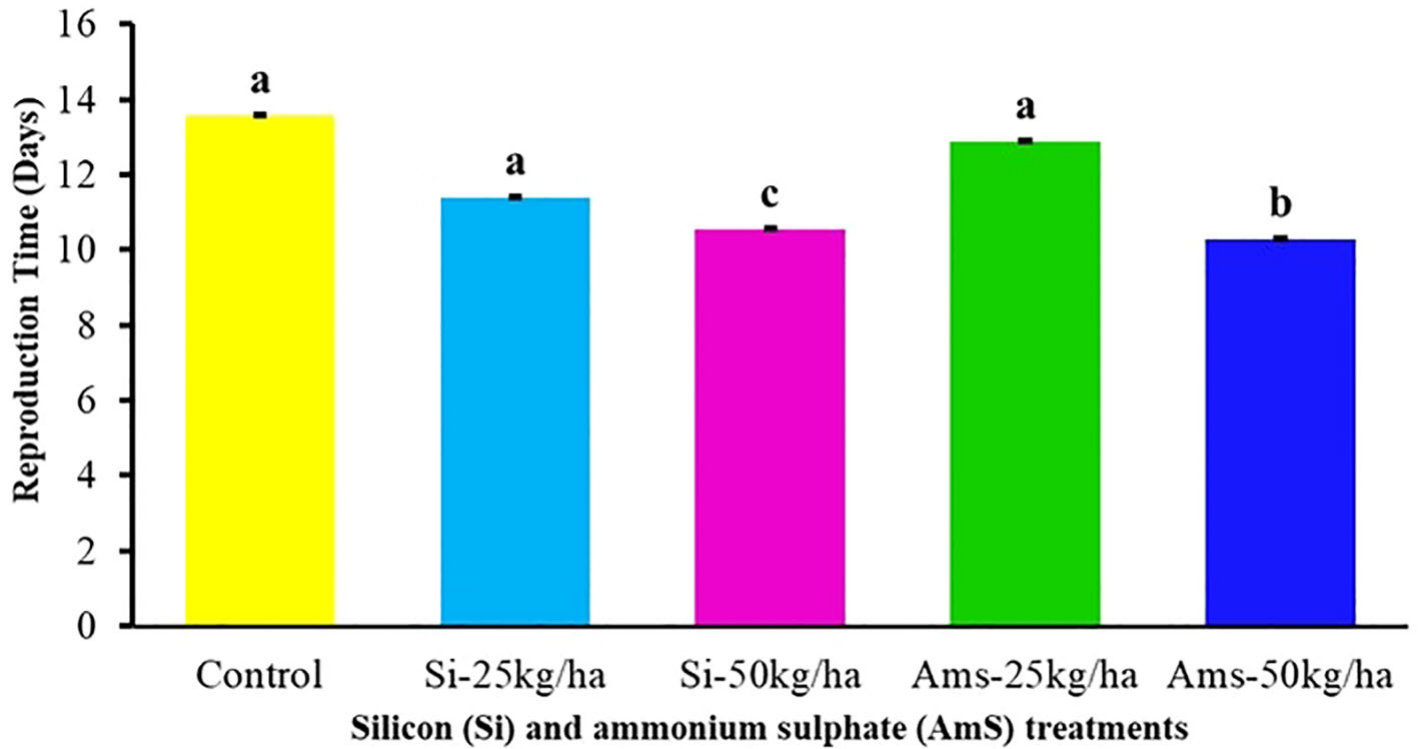
*Myzus persicae* reproduction time (days) after soil treatment with silicon (Si) and ammonium sulphate (Ams). Means (± SD, *n* = 10) with different letters differ significantly (*p* < 0.05).

**Figure 8:**
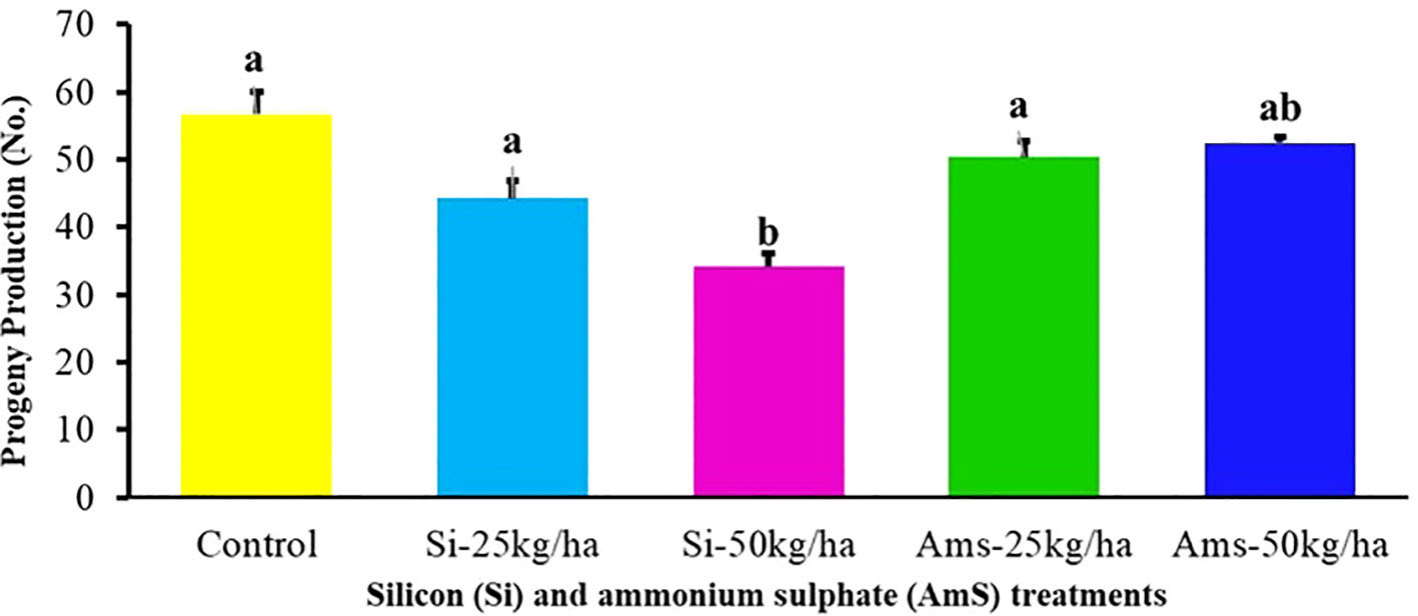
Progeny production (No.) of *Myzus persicae* reared on *Brassica napus* host plants subjected to soil-incorporated treatments of silicon (Si) and ammonium sulphate (Ams) in a herbivore-deterrence bioassay. Bars represent mean ± SD (*n* = 10). Different lowercase letters indicate statistically significant differences among treatments (Tukey’s HSD test, *p* < 0.05).

**Figure 9:**
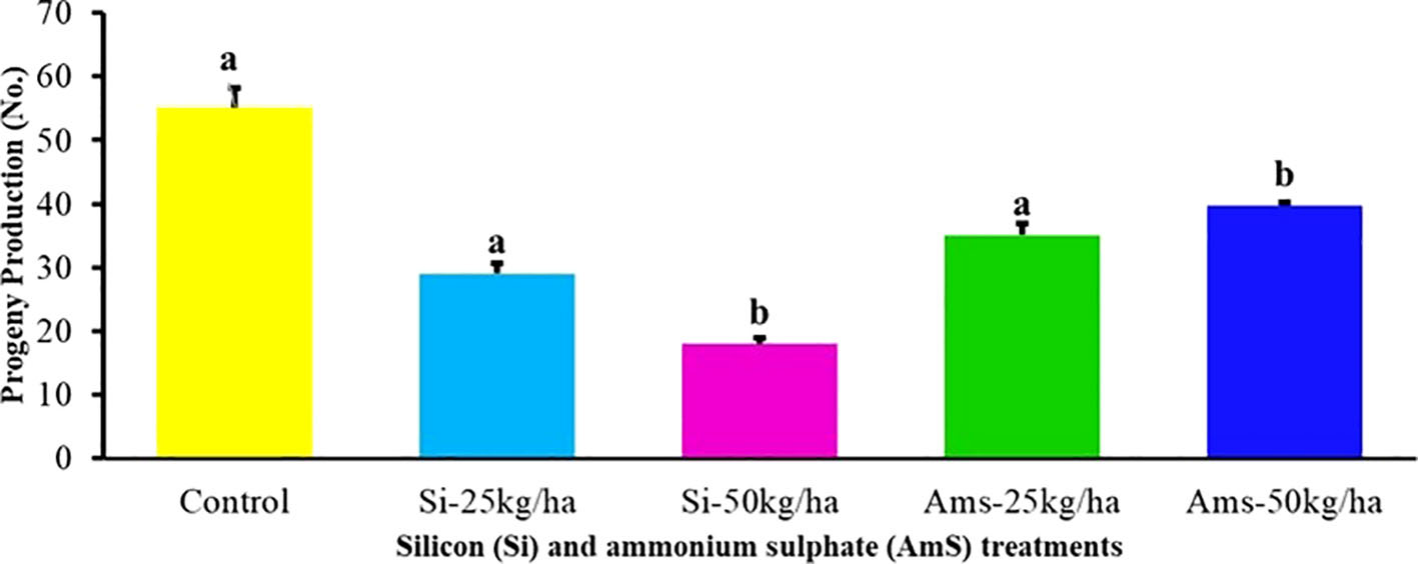
Effect of soil-incorporated silicon (Si) and ammonium sulphate (Ams) on immature of *Myzus persicae* transforming into adults in a herbivore-deterrence assay. Values are mean ± SD (*n* = 10). Different letters denote significant differences (*p* < 0.05).

**Figure 10:**
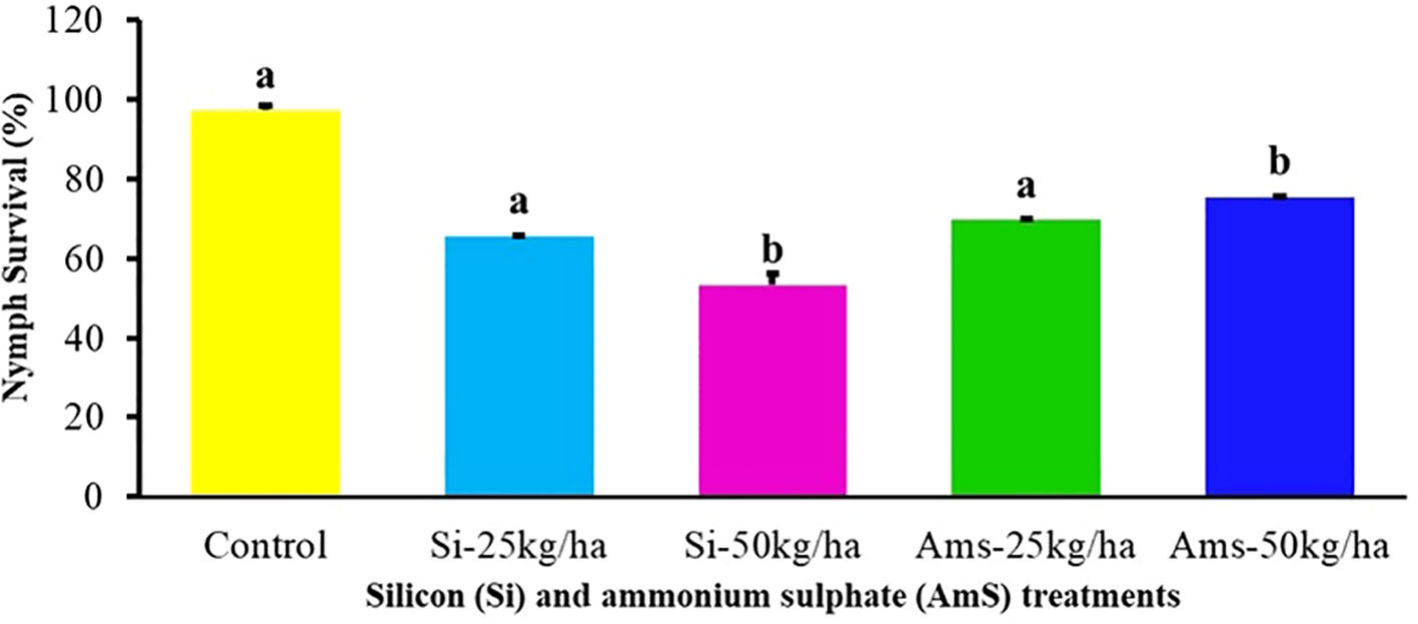
The nymph survival (%) of *Myzus persicae* as influenced by soil amendments of silicon (Si) and ammonium sulphate (Ams) in a herbivore-deterrence experiment. Data are presented as mean ± SD (*n* = 10). Bars annotated with different lowercase letters are significantly different (*p* < 0.05).

The developmental period of *M. persicae* was significantly (F_4, 54_ = 5.4, *p* < 0.01) affected by both treatments (silicon-Si and ammonium sulphate-Ams) relative to the control (**Figure 6**). Results depicted that the maximum time (10.8 days) for the development of *M. persicae* was observed in 50 kg ha^-1^ Si (23.5%) followed by 25 kg (17.6%). The Ams treatments were ineffective relative to the control. Similar trend was observed in reproduction time of aphids which were significantly (F_4, 54_ = 5.9, *p* < 0.001) influenced by Si and Ams as compared to the control (**Figure 7**). The results showed that maximum declined in reproduction time found in Ams-50 kg ha^-1^ (25.01%) followed by Si-50 kg ha^-1^ (21.7%). The progeny production was maximum influenced by Si-50 kg ha^-1^ (40.3%) followed by Si-25 kg ha^-1^ (23%) and Ams-50 kg ha^-1^ was slightly effective showing 8.1% reduction only; however, Ams-25 kg showed 13.7% reduction in *M. persicae* progeny (**Figure 8**). Immature becoming adults were also statistically significant (F_4, 54_ = 43.3, *p* < 0.001) in impact over the control treatments (**Figure 9**). Highest conversion was seen in the control followed by Ams-50 (35.6%) and 25 kg ha^-1^ (28.1%). However, least conversions were there in Si 25 (45.3%) and 50 kg ha^-1^ (66.04%) over the control. The nymphal survival percentage also displayed statistically significant difference (F_4, 54_ = 51.15, *p* < 0.001) from control treatment (**Figure 10**). The results depicted that minimum nymph survival percentage was observed in Si-50 kg ha^-1^ (47.7%) followed by Ams-50 kg ha^-1^ (69.1%) and Si-25 kg ha^-1^ (63.8%) whereas almost all nymphs (98.3%) were survived in case of control treatment.

### 3.3 Soil amendments experiment

**Figure 11:**
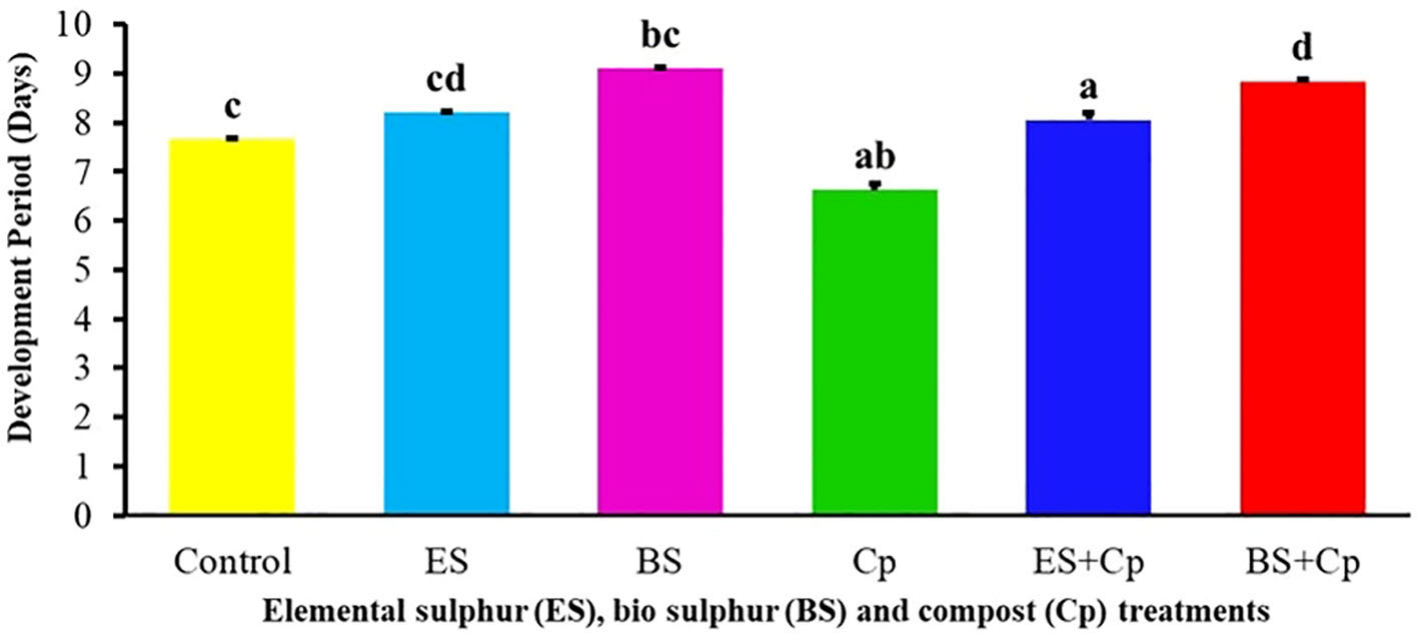
Effect on developmental period (days) of *Myzus persicae* under soil amendments experiments due to soil-incorporated treatments of elemental (ES), bio sulphur (BS), compost (Cp), and their mixtures. Bars indicated the data (Means ± SD, *n* = 10) having annotated different lowercase letters (Statistical significance *p* < 0.05).

**Figure 12:**
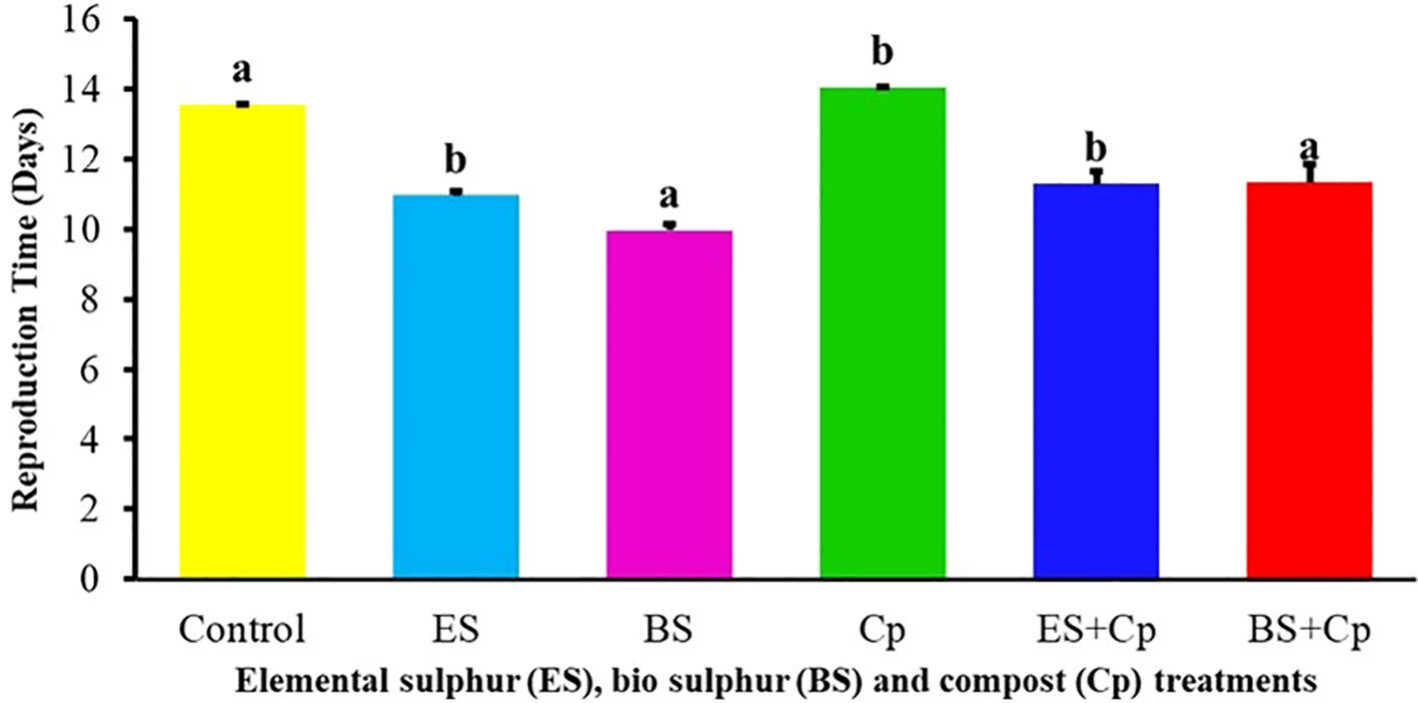
Effect of soil applications of elemental sulphur (ES), bio sulphur (BS), compost (Cp), and their combinations on the reproduction time (days) of *Myzus persicae*. Data represent mean ± SD (*n* = 10). Different lowercase letters indicate statistically significant differences (*p* < 0.05) among treatments.

The results revealed that the developmental period of *M. persicae* was delayed significantly (F_5, 54_ = 54.7, *p* < 0.0001) as compared to the control for BS formulation by 12.5%, and to a similar level by BS+Cp relative to the control (**Figure 11**). Addition of Cp reduced this parameter substantially. Other treatments were ineffective. Parallel trends were seen for the reproduction time of aphids that were significantly affected by the application of sulphur formulations (ES: 22.7%, BS: 27.4%) and their mixture with compost (ES+Cp: 21.5% ES+Cp: 21.8%), compared to the control (**Figure 12**). Effects of compost were not different from those of control plants.

**Figure 13:**
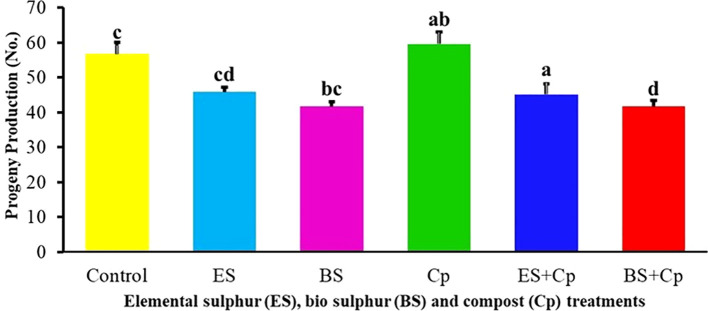
Progeny production (No.) of *Myzus persicae* reared on *Brassica napus* subjected to soil treatments of elemental sulphur (ES), bio sulphur (BS), compost (Cp), and their combinations in a soil amendments assay. Bars represent mean ± SD (*n* = 10). Different lowercase letters indicate statistically significant differences among treatments (Tukey’s HSD test, *p* < 0.05).

**Figure 14:**
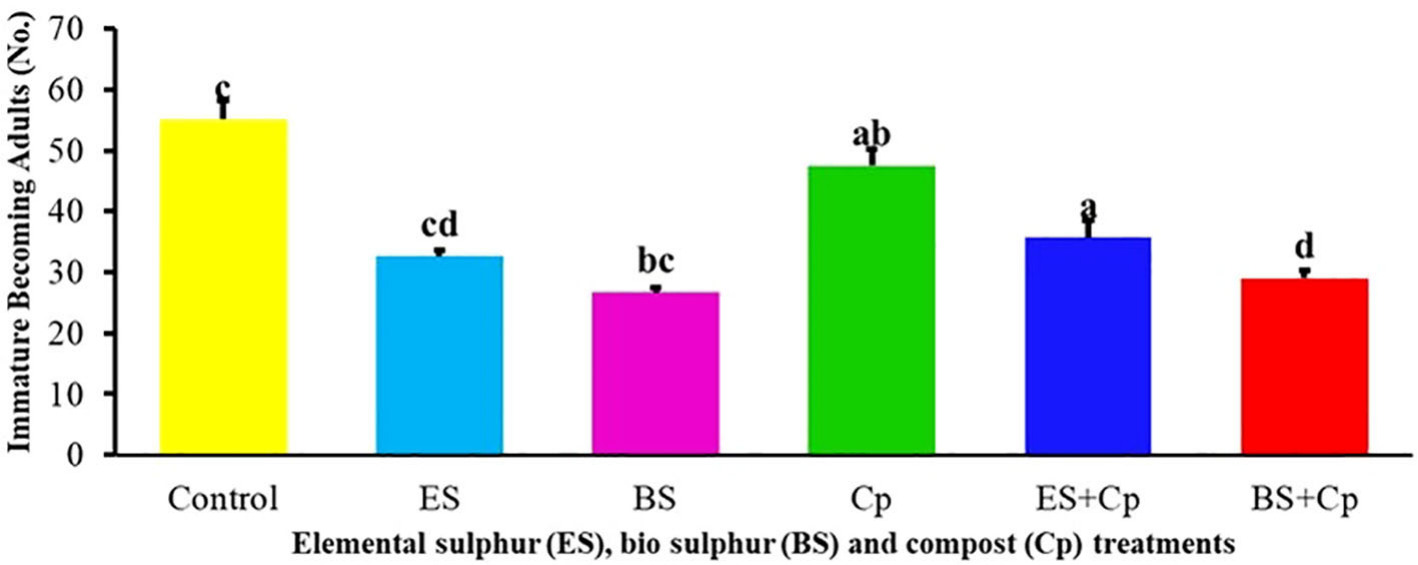
Conversion of immature to adults of *Myzus persicae* under soil amendments experiments due to elemental sulphur (ES), bio sulphur (BS), and compost (Cp) treatments. Values are mean ± SD (*n* = 10). Different letters denote significant differences (*p* < 0.05).

**Figure 15:**
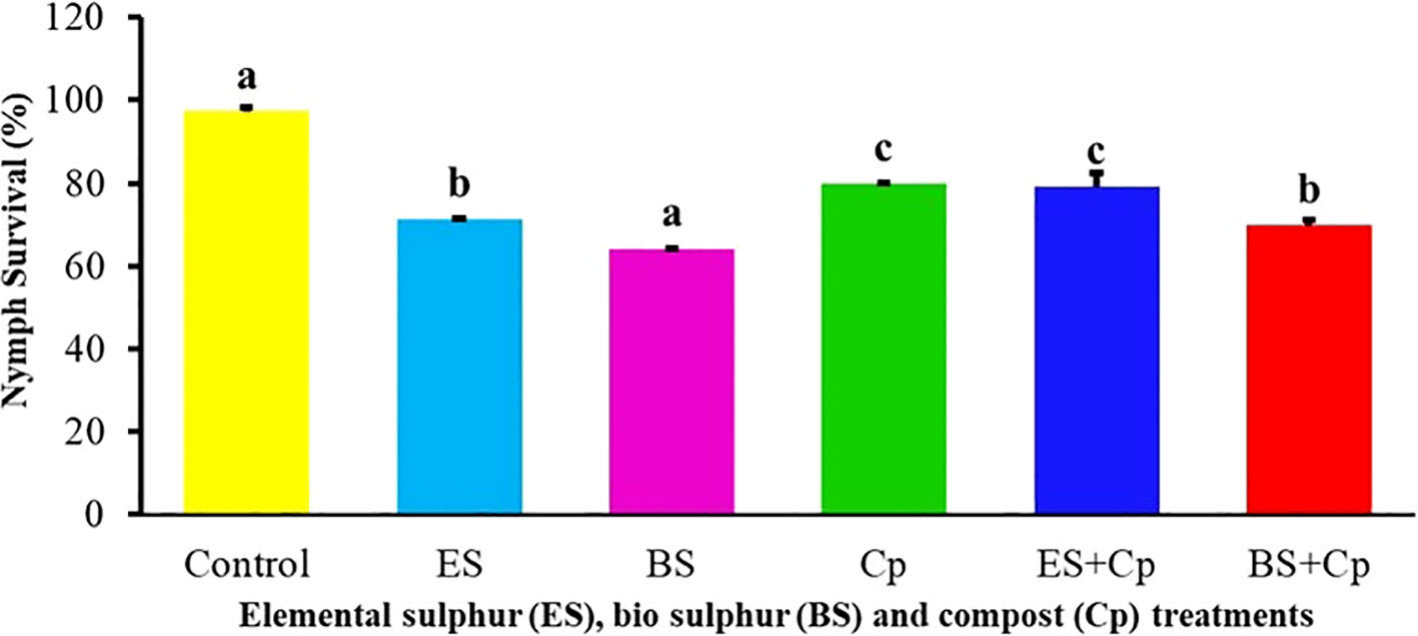
Nymph survival (%) of *Myzus persicae* under soil amendments experiments due to elemental sulphur (ES), bio sulphur (BS), and compost (Cp) treatments. Bars indicated mean values ± SD (*n* = 10) and annotated different lowercase letters showed statistical significance (*p* < 0.05).

Progeny production also found significantly different across treatments (F_5, 54_ = 124.5, *p* < 0.0001) (**Figure 13**). The BS+Cp had the lowest progeny (23.7%) followed by BS (22.1%), ES+Cp (17.03%), and ES (17.1%). The conversion of immature aphids was also found significantly influenced (F_5, 54_ = 141.3, *p* = 0.006) by the treatments (**Figure 14**). Here, BS was more efficiently reduced the conversion (59.3%) as compared to control treatment followed by BS+CP (49%), ES (44.2%), ES+Cp (42.9%), and Cp (23.06%). The nymphal survival percentage also displayed statistically significant difference from control treatment (F_5, 54_ = 66.18, *p <* 0.0001) (**Figure 15**). The results depicted that minimum nymph survival percentage in BS (41%), followed by ES (35.5%) and BS+CP (35.3%) compared with the control. Individual and combined use of compost with either sulphur formulation did not give any additional gain.

## 4 Discussion

### 4.1 Foliar interventions

The Impact of SA and CA was significant on different parameters of *M. persicae*. The results showed that 1 mM SA showed maximum efficacy to increase the developmental time and decrease the reproduction period over other treatments. Khoshfarman-Borji et al. (12), along with Yali and Sattari-Nassab (23) observed the similar impacts on aphids (*Brevicoryne brassicae*) and their results were in-line with our studies. They observed that the SA and CA negatively affected the survival and developmental time of aphid including production of aphid progenies and the transformation of immature into adult aphids. Higher concentrations were more effective in reducing pest fitness as they were reported to activate toxic biochemicals (phenolics and glucosinolates) and plant defense pathways, maintain redox balance, and to generate physical defense in plants (12, 19–21).

Nasab et al. (24) also recorded higher concentrations of inducers to be more effective while inducing the resistance in *B. napus* plants. Application of these plant elicitors induced the resistance in plants by modulating the biochemicals and antioxidant defense systems in plants that were activated to reduce the progeny and colony development of the aphids (10). Among defense chemicals, phenolic and glucosinolates are thought to play an important role to negatively regulate the aphid population (15).

This study also disclosed the further information about citric acid that can enhance the plant defense system against biotic stress particularly aphids. The results also clued about the trans-generational effects of SA and CA regarding the less conversion of neonates into adults and progeny survival. However, studies are needed to confirm the role of the inducers on biocontrol agents under diverse agro-climatic zones.

### 4.2 Herbivore-deterrence

Our experiments, first time explored the impacts of Si and Ams on *M. persicae*. Progeny development and other biological attributes of *M. persicae* were significantly affected by both nutrients, i.e., silicon (Si) and ammonium sulphate (Ams). Abbas et al. (9) found similar conclusion while working on potassium silicate and stated that treating the canola plants with Si had a substantial impact on different biological parameters and total populations of aphids while activating the plant defense. Similar results were also observed by de Oliveira et al. (25) and found that plants treated with Si showed significant resistance against aphids and other insect pests. Teixeira et al. (26) also found a notable reduction in aphid biological and life history characteristics after silicon treatments.

Application of nutrients regulated aphid survival by reducing the number of aphid progenies. El-Naggar et al. (27) also confirmed that less nymphs and adults were survived on Si-treated plants as compared to the control. Abbas et al. (9) González-Hernández et al. (14) de Oliveira et al. (25), and Teixeira et al. (26) El-Naggar et al. (27) also found that Si and Ams significantly declined the aphid and other sucking insect population on brassica crop. Since Ams is a common fertilizer for several brassica crops, its role to reduce aphid colonization and population were also confirmed under field conditions (14). Si and Ams are vital minerals that may increase plant resilience, enabling the plants to withstand harmful effects of insects by regulating morpho-physiological changes in plants especially the activation of glucosinolates and phytoliths (26, 27). Besides, 25 and 50 kg doses, these nutrients must be tested at other field recommended doses to ascertain their role in diverse cropping system.

### 4.3 Soil amendments

Elemental sulphur (ES), Bio Sulphur (BS), compost (CP) and their combinations had inconclusive effects. Results regarding the individual use of compost were not encouraging, however; its combination with both sulphur formulations provided additional gains. Besides glucosinolates that are activated in Brassica crops in response to sulphur application, role of rhizobacteria, phenolic chemicals, and plant nutrients found important (24). It is important to note that in our previous findings bio sulphur improved the performance of *B. brassicae*; however, it reduced the performance in case of *M. persicae*. This might happen because *B. brassicae* is a specialist herbivore that could effectively manipulate the bio sulphur-mediated plant defense, which may be lacking in *M. persicae*. Therefore, transcriptomic studies on aphid saliva and plants are necessary to decipher this outstanding variation of two different aphid species on same host plant under similar treatment conditions.

Conclusive reason behind the performance of inducers include the activation of pathogenesis related proteins and phenyl ammonia lyase to activate salicylic and jasmonic acid defense pathways (11, 12, 20). Herbivore-deterrence treatments reduced aphid biological fitness due to nutrient-specificity where Ams treatment may reduce insect attributes by increasing the level of defense chemicals particularly glucosinolates and phenolic compounds mediated by SAA (14). Si treatment works well by impeding aphid stylet entry (25) and reducing food digestion through silica layer buildups in leaf tissues (9, 26). Soil amendments may perform well due to presence of sulphur which is an important ingredient to improve plant glucosinolates defense (15) while compost functions to reduce insect performance through the activation of proteolytic enzymes in insect bodies while improving plant health (16). These treatments are required to be tested under field condition along with proper assessment of plant morphometric characters, yield parameters and plant defensive chemical enhancements as compared to the control.

## 5 Conclusion

Applying foliar and soil interventions, specifically nutrients and sulphur, altered the biological fitness of *Myzus persicae*. These results support the initial hypothesis and highlight the role of such treatments in sustainable pest management. The effectiveness of these interventions stemmed from their ability to activate defense genes and enhance biochemical levels while simultaneously boosting plant vigor. This approach offers an ecologically safer strategy for integrated pest management across various insects and crops. However, fully incorporating these findings into the breeding of aphid-resistant cultivars will require a deeper understanding of the underlying molecular mechanisms driving plant resistance.

## Data Availability

The original contributions presented in the study are included in the article/**Supplementary Material**. Further inquiries can be directed to the corresponding author/s.
