# Supplementary material for: Soil nutrition and foliar intervention in Brassica napus (L.) to impair biological fitness of green peach aphid (Myzus persicae Sulzer)
Source: Front Insect Sci. 2026 Jun 26;6:1853500. doi: 10.3389/finsc.2026.1853500 (PMC13350061; doi:10.3389/finsc.2026.1853500)
Supplement: Supplementary file 1 [file Table1.docx]

| Table S1. Specifications of inducers, nutrients, and sulphur treatments. | | |
| --- | --- | --- |
| **Treatments** | **Specifications** | **Suppliers** |
| Salicylic Acid | Purity 99.0 % | Sigma Aldrich, St. Louis, MO |
| Citric Acid | Purity 99.5 % | Sigma Aldrich, St. Louis, MO |
| Ammonium Sulphate | Nitrogen: 21%, Sulphur: 24% | Engro Fertilizers, Pakistan |
| Silicon (SiO_2_) | Silicon: 46.7%, O_2_: 53.3%, Purity: 99.0% | Merck, Darmstadt, Germany |
| Elemental Sulphur | Sulphur: 99.5% (Powder), Inert: ≤1% | Faisalabad, Pakistan |
| Bio Sulphur | Sulphur: 70%, NPK: ~2.5%, 1.5%, 3% | ISES, UAF |
| Compost (g kg^-1^) | Carbon: 210.5, Nitrogen: 17.1, P: 3.01, pH: 6.43 | ISES, UAF |
